# Medical Yoga for Patients with Stress-Related Symptoms and Diagnoses in Primary Health Care: A Randomized Controlled Trial

**DOI:** 10.1155/2013/215348

**Published:** 2013-02-26

**Authors:** Monica Köhn, Ulla Persson Lundholm, Ing-Liss Bryngelsson, Agneta Anderzén-Carlsson, Elisabeth Westerdahl

**Affiliations:** ^1^Nora Health Care Centre, Örebro County Council, 713 31 Nora, Sweden; ^2^Department of Occupational and Environmental Medicine, Örebro University Hospital, 701 85 Örebro, Sweden; ^3^Centre for Health Care Sciences, Örebro University Hospital, P.O. Box 1324, 701 13 Örebro, Sweden; ^4^School of Health and Medical Sciences, Örebro University, 701 82 Örebro, Sweden

## Abstract

An increasing number of patients are suffering from stress-related symptoms and diagnoses. The purpose of this study was to evaluate the medical yoga treatment in patients with stress-related symptoms and diagnoses in primary health care. A randomized controlled study was performed at a primary health care centre in Sweden from March to June, 2011. Patients were randomly allocated to a control group receiving standard care or a yoga group treated with medical yoga for 1 hour, once a week, over a 12-week period in addition to the standard care. A total of 37 men and women, mean age of 53 ± 12 years were included. General stress level (measured using Perceived Stress Scale (PSS)), burnout (Shirom-Melamed Burnout Questionnaire (SMBQ)), anxiety and depression (Hospital Anxiety and Depression Scale (HADS)), insomnia severity (Insomnia Severity Index (ISI)), pain (visual analogue scale (VAS)), and overall health status (Euro Quality of Life VAS (EQ-VAS)) were measured before and after 12 weeks. Patients assigned to the Yoga group showed significantly greater improvements on measures of general stress level (*P* < 0.000), anxiety (*P* < 0.019), and overall health status (*P* < 0.018) compared to controls. Treatment with medical yoga is effective in reducing levels of stress and anxiety in patients with stress-related symptoms in primary health care.

## 1. Introduction

Stress is a major public health problem and may lead to reduced quality of life, lower work efficiency, increased human suffering, and increased consumption of health care. Stress and stress-related diagnoses are causes for high sick-leave rates [1]. 

In primary health care, a growing number of patients are suffering from stress-related symptoms and diseases. Stress as a phenomenon is difficult to define, and the term encompasses symptoms such as cognitive problems, fatigue, and disrupted sleep [2]. More and more people are experiencing stress as a serious problem often due to a burdensome work situation but also due to lack of stimulation and meaningful employment. Today, the lack of time for rest and recovery seems to be a bigger health problem than physical and mental strain at work [3]. 

Other stress-related problems include chronic pain and psychiatric symptoms such as anxiety and depression. People with higher self-rated stress are more likely to develop depression when negative life events occur, compared with those who rate lower stress levels [4]. Depression is one of the most common causes of ill health, lost productivity, and disability. Stress, depression, and anxiety are factors that adversely affect people's perception of well-being [5]. Fatigue and sleep disturbances are common symptoms [6]. Even minor disturbances in the quantity of sleep take their toll in terms of mental function [7]. Stress is also one of the factors leading to musculoskeletal disorders. Chronic pain often has serious social and psychological consequences affecting the quality of life and daily activities [8, 9]. Physical activity is highlighted as a key component to enhance the human experience of well-being. Regular exercise helps people to better overcome stress [5]. Yoga is used for wellness care in large parts of the world and has recently also been introduced as therapy in health care [10–13]. 

There are numerous schools or types of yoga, and most of them have the following key elements: breathing exercises (pranayama), postures (asana), and meditation.Medical yoga derives from the classic Kundalini yoga with its origins in northern India and Tibet. Yoga is specifically characterized to achieve mental, emotional, and physical effects. Medical yoga has been designed to suit people who are severely ill but who can practise yoga without the risk of worsening their condition. A yoga class consists of a number of positions, movements, breathing techniques, mantras, and meditation. The starting position can be sitting, lying, or standing, and the movements are generally slower than in traditional forms of yoga. Several Swedish and international studies have shown measurable psychological, and physiological effects after regular yoga exercise [13–16]. 

These studies have primarily examined therapeutic benefits to individuals in private yoga practice or in the inpatient setting. However, no scientific studies have examined the effects of medical yoga on stress-related symptoms in patients in primary care. The purpose of this study is to investigate whether the treatment with medical yoga has effects on the stress levels, anxiety, depression, pain, sleep, and quality of life among primary care patients with stress-related symptoms and diagnoses.

## 2. Material and Methods

### 2.1. Design and Patients

**Figure 1:**
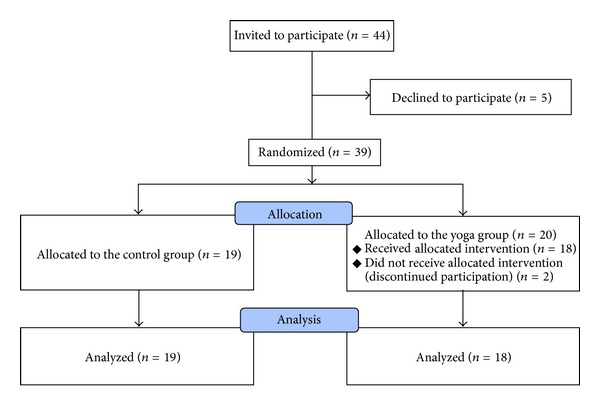
Flow chart outlining participation in the study.

The study design was a randomized controlled trial. A total of 44 patients with stress-related symptoms or diagnoses in a primary health care centre in Sweden were invited to participate in the study. The patients had sought treatment at the primary health centre during the previous 6 months. Patients suitable for inclusion were identified by the general practitioners, counselor, district nurses, or physiotherapists at the patient's first visit or at an ongoing contact at the primary health care centre. One of the two physiotherapists, responsible for the study, then invited the patients to participate in the study. Five patients declined participation at the time of the invitation, and after randomization, two more patients discontinued participation. A total of 37 patients were finally analyzed in the study (Figure 1).

The inclusion criteria were self-reported symptoms of stress as well as a stress-related diagnosis. A versatile definition of stress-related symptoms was applied. The stress-related symptoms included fatigue, insomnia, anxiety, depression, hypertension, or musculoskeletal discomfort in the neck and shoulders. Exclusion criteria were the inability to understand instructions, interpretation needs, or physical or mental inability to carry out the medical yoga exercises, such as mental retardation or dementia and severe physical or mental illness. Informed consent was obtained from each patient. The research procedure was in accordance with the Helsinki Declaration, 2008, and the study was approved by the Regional Ethical Review Board in Uppsala, Uppsala, Sweden (2011/043). The trial has been registered with ClinicalTrials.gov, a service of the U.S. National Institutes of Health: NCT01604707.

The patients were randomized to a yoga group performing medical yoga training during a 12-week period or a control group performing no medical yoga. All patients were also offered a standard treatment at the primary health care centre. The standard treatment comprised of prescribed pharmacological treatment, individual physical activity, or individual consultations with a nurse, psychologist, physiotherapist, or counselor. A computer-generated list was used for the randomization. Group allocation was prepared in sequentially numbered, sealed opaque envelopes, by an independent person. One of the two physiotherapists responsible for the study performed the randomization. Written informed consent was obtained prior to the randomization.

### 2.2. Study Groups

The patients assigned to the yoga group participated in medical yoga therapy once a week over a 12-week period from March to June, 2011. Yoga treatment was performed as a group training (two groups/week, with eleven patients in each group), with classes lasting about 60 minutes and being led by a physiotherapist who was a certified yoga instructor in medical yoga. The yoga class consisted of a number of postures and stretching exercises, breathing techniques, mantras, and meditation. The yoga was performed in lying and sitting positions. The yoga sessions began with slow breathing exercise in lying (about 10–15 minutes). Thereafter, gentle movements of the whole body and stretching exercises in a sitting position were performed. Each exercise was performed 1–3 minutes in separate positions during a session of 20–30 minutes. After these exercises, a long relaxation (Savasana) for 10–15 minutes took place. The session ended with 5–10 minutes of meditation. The training was performed in accordance with instructions from the Institute for Medical Yoga, Stockholm, Sweden (http://www.medicinskyoga.se/). 

### 2.3. Outcome Measures

Baseline measurements in all patients were performed the week before the study period. Measurements were repeated, after the 12 weeks of medical yoga treatment. The data collection was performed at the primary health care centre. The Swedish version of the Perceived Stress Scale (PSS) was used to measure the general stress level. The instrument was developed by Cohen et al. [17] and translated into Swedish by Eskin and Parr [18]. The PSS includes 14 questions on perceived stress in the previous month. Responses are given on a Likert-type scale ranging from 0 (never) to 4 (very often). The total score is calculated with a possible score range of 0–56. A higher score reflects greater perceived stress. Assessment of PSS after the study period was performed by telephone 2 months after the completion of the study.

Symptoms of burnout were assessed with a translated version of the Shirom-Melamed Burnout Questionnaire (SMBQ) [19]. The instrument consists of 22 questions graded from 1 (almost never) to 7 (almost always) and shows four dimensions of burnout: emotional and physical exhaustion, listlessness, tension, and cognitive weariness. A higher score indicates a higher level of burnout. A critical reference value is set at 3.75 [19, 20]. 

The Swedish version [21] of the Hospital Anxiety and Depression Scale (HADS) [22] was used to assess anxiety and depression. The scale consists of 14 items measuring symptoms on a 4-point Likert scale, with a score range of 0–3, a higher rating indicating a higher state of anxiety or depression. The limit for moderate symptoms is 8 points; for clinically relevant signs, the range is 10–16 points and the threshold for severe anxiety or depression is 16 points or more.

An unmarked, continuous, 10 cm horizontal visual analogue scale, ranging from 0 (no pain) to 10 (the worst imaginable pain) was used to assess the patients pain before and after the study period. The pain scale was supplemented with a pain drawing of the body in which the patient filled in the areas of the body that hurt.

Insomnia was assessed using the Insomnia Severity Index (ISI) [23]. The questionnaire consists of seven questions, with problems estimated on a 4-point Likert-type scale. Values between 8 and 14 points indicate some problems with sleep, while scores between 15 and 21 points indicate a moderate sleep disorder, and more than 22 points are regarded as indicating severe insomnia. 

To measure the patients' perception of overall health status, we used the Euro Quality of Life VAS (EQ-VAS) ranging from 0 (worst imaginable health) to 100 (best imaginable health) [24]. Furthermore, heart rate and peripheral oxygen saturation (SaO_2_) were measured with a pulse oximeter (Rad-5v; Masimo, Irvine, CA, USA) and blood pressure with an automatic blood pressure cuff (TriCUFF; AJ Medical, Lidingö, Sweden). Thoracic excursions were measured using a tape (marked in mm) around the circumference of the chest to give a measurement of chest expansion or mobility. Thoracic excursions were measured at the level of the xiphoid process, performed with the patients standing with their hands placed on their head and given the instruction “breathe in maximally and make yourself as big as possible” and “Breathe out maximally and make yourself as small as possible.” [25] 

After the intervention, the patients in the yoga group were asked whether or not they had felt any benefit and/or any discomfort (yes/no) of the medical yoga training.

### 2.4. Statistical Analysis

 All data were collected and analyzed using SPSS version 14.0 (SPSS Inc., Chicago, IL, USA). The primary outcome measure was the general stress level as measured with the PSS. Including 18 patients per group would yield 80% of power (alpha = 0.05) to detect a difference of 7 units between the groups, assuming a standard deviation (SD) of 7.35, calculating from SD as described by Brinkborg et al. [26]. This difference is assumed by the authors to be of clinical relevance. Since dropout was anticipated to be 15–20%, we included another four patients in each group, bringing the total number of patients in the study to 44.

The differences between baseline and after treatment scores were calculated for the outcome measures and were compared between the groups. For the comparison of perceived stress, anxiety, depression, pain, insomnia, and overall health status between the two groups, a nonparametric test, the Mann-Whitney *U* test, was used. For the comparison of heart rate, oxygen saturation, blood pressure, and thoracic excursions between the two groups, an unpaired Student's *t*-test was used. All results refer to two-sided tests, and *P* values less than 0.05 were considered significant.

## 3. Results

Of 44 eligible patients with stress-related symptoms or diagnoses seeking care at a primary health care centre, 39 patients agreed to participate in the study. Two women in the yoga group later chose to withdraw from the study, as shown in the flow chart in Figure 1. The reasons for withdrawal were family related or recently acquired acute disease. In total, 37 patients (34 female and 3 male) were included and evaluated before and after the 12-week study period. Mean age in the sample was 53 ± 12 years. Demographic data at baseline (Table 1) did not significantly differ between the two groups. 

The patients often indicated several symptoms or health complaints, but the predominant symptom given in the patients' medical records was pain (*n* = 12), stress (*n* = 10), anxiety (*n* = 5), hypertension (*n* = 3), depression (*n* = 2), migraine headaches (*n* = 2), dizziness (*n* = 1), and fatigue (*n* = 2). Diagnoses and their codes, based on the Swedish classification system, reported in the patients' medical records, were pain (R52), somatoform pain syndrome (F454), generalized anxiety disorder (F411), myalgia (M791), hypertension (I10), migraine (G43), stress (F439P), depressive episode (F32), anxiety disorder (F419P), recurrent depressive disorder (F33), life problems (Z73), panic disorder (F410), fatigue (F438), and supraspinatus syndrome (M751). The symptoms were either new onset (within 3 months) or long term (9 ± 8 years, range approximately 1–41 years). At baseline, 21 of the patients were employed, 10 were on sick leave, and 6 were retired for reasons of age (Table 1). In total 12 patients in the medical yoga group had full compliance and 6 patients were absent at one or two times of the 12 treatment occasions. 

### 3.1. Stress and Burnout Symptoms

 Mean baseline scores on the PSS (possible range 0–56) were 34 ± 11 in the control group and 36 ± 10 in the yoga group, with no significant difference between the groups. A significantly greater improvement in PSS scores was seen in the yoga group compared with the control group (*P* < 0.000) after the completion of the study (Table 2). 

Burnout was high in both groups at baseline, with 30/37 patients (81%) reporting scores over the reference limit value of 3.75, indicating burnout symptoms [19, 20]. No significant differences between the control group and the yoga group were shown with regard to changes in self-reported burnout symptoms after the study period (*P* < 0.412) (Table 2).

### 3.2. Anxiety and Depression

 Mean anxiety/depression scores at baseline were comparable between groups. According to the HADS score, 65% of the patients had clinically relevant signs of anxiety, and 32% had clinically relevant signs of depression at baseline. After the 12-week intervention, there was a significant difference between the groups regarding the decrease in anxiety/depression symptom scores measured as total HADS score, favouring the yoga group (*P* < 0.047) (Table 2). The subscales showed that the decrease in anxiety symptoms was significantly more pronounced in yoga group than in control group (*P* < 0.019), but there was no significant difference between groups regarding the subscale for depression (*P* < 0.123) (Table 2). 

### 3.3. Insomnia

Regarding insomnia, 16 (43%) of the patients scored ≥14 points on the ISI, indicating moderate or severe sleep disturbance at baseline. After the medical yoga intervention, the values were unchanged in the control group, and had improved in the yoga group (Table 2), but when comparing the change from baseline between the groups no statistically significant difference was present.

### 3.4. Pain

Mean values for VAS were before study 4.0 ± 2.3 in the control group and 2.8 ± 2.7 in the yoga group. After 3 months, the pain had decreased in both groups (control group 3.5 ± 1.9 and yoga group 2.2 ± 2.5). The reduction was comparable between the control group and yoga group after the study period (*P* = 0.871). Data on pain, compiled from the pain drawings of the body, are given in Table 3.

### 3.5. Overall Health Status and Subjective Experiences of Yoga Treatment

Change in perception of overall health status, measured by EQ-VAS, showed significant differences between the groups after the 12-week period (*P* < 0.018), as presented in Table 2. After the yoga intervention, all (*n* = 18) patients in the yoga group reported some subjective benefit of the treatment, described as contact with inner feelings and emotions, experience of peacefulness, and knowledge of how-to-use breathing technique. Discomfort related to the medical yoga was reported by 5 (28%) patients. The adverse experiences given were related to the strain experienced with contact with one's inner feelings as well as to the burden of having to take care of oneself. Some patients mentioned that the yoga period was too short.

### 3.6. Thoracic Excursions and Oxygenation

There was a significant difference between the two groups in change in thoracic mobility as measured by thoracic excursion measurements (*P* < 0.007). The yoga group had increased the difference in size between inspiration and expiration from 3.3 ± 1.6 to 4.2 ± 2.0 cm, while the control group had decreased the difference in circumference size from 3.6 ± 3.7 to 2.7 ± 2.0 cm. Values for heart rate, SaO_2_, and blood pressure are presented in Table 4. No significant differences were noted between the groups, before or after the medical yoga intervention.

## 4. Discussion

The major findings in this study were that medical yoga, performed as 12 weeks of group training in a primary health care setting, was effective in reducing levels of stress and anxiety in patients with stress-related symptoms. To our knowledge, this is the first study to evaluate the effect of medical yoga in patients with stress-related symptoms and diagnoses in a primary health care setting. Many persons are on sick leave because of stress-related causes, and an increasing number of patients seek primary health care because of stress-related symptoms [2]. It is therefore important to focus on interventions that are possible to perform in this specific setting [1]. Stress as a phenomenon can be difficult to define, and the symptoms could vary considerably between individual patients. Accordingly, the improvement caused by the medical yoga intervention could be expected to vary in different symptom areas, as presented in this study.

The PSS was used for measuring general stress level in the patients. After the yoga treatment, the patients in the yoga group had significantly reduced their levels of stress, in comparison with patients in the control group. As the PSS measures degree to which an individual experiences their life as unpredictable, uncontrollable, and overloaded [17, 18], it is possible that yoga provides the patients with strategies to deal with stress-related symptoms and to be in better control of their life, which in turn reduces levels of stress. 

In spite of the decreased level of stress in the yoga group, there were no statistically significant differences between the two groups with regard to depression, as measured with the HADS. The reason for this is unknown, but similar results were found in a previous study, where women with depression and anxiety were treated with yoga over 12 weeks [27]. It is possible that 12 weeks of training is a too short period of time to have an effect on depression, as suggested by Javnbakht et al. [27]. However, the level of anxiety was significantly decreased in the yoga group, compared with the control group. Anxiety has been defined as an emotion that is a response to an undefined or unspecific threat perceived by the individual. It is possible that yoga provides tools for dealing with such emotions. This in turn could make the patients more secure and increase their self-esteem, which may decrease stress [1]. According to the medical records, anxiety was the predominant symptom for only 5 (13%) patients in the present study. However, according to HADs anxiety score, 65% of the patients had clinical relevant signs of anxiety. In a previous systematic review of yoga as a treatment for anxiety, positive results due to yoga were reported. However, methodological inadequacies were identified in several of the included studies [28]. In none of the included studies, HADS was used as a primary outcome measure. The result in the present study is in line with the previous studies as reported by Kirkwood et al. [28], although using another outcome measure for anxiety. The HADS has been reported to be an easily administered measure with high internal consistency (Cronbach's *α* 0.79–0.94) in clinical populations [29].

After the study periods, the patients in the yoga group had significantly improved scores in overall health status, measured by the EQ-VAS, compared with the patients in the control group. When interpreting this finding, it would have been valuable to have some additional information on what aspects the individual patients take into account when scoring their overall health status. It would also have been interesting to correlate these findings to the perceived stress level.

There was a significant difference in thoracic excursion after the intervention between groups. In patients in the yoga group, thoracic excursions were significantly increased compared to the control group. Stress may lead to muscular tension, which in turn can affect the thoracic mobility and respiratory pattern. The exact mechanism behind the increased thoracic excursion in the present study is unknown, but it is reasonable to believe that by reducing stress, the yoga intervention had an indirect effect on the muscular tension in the thorax area. Various yoga positions make the muscular tissue more lean and the joints more flexible, which in turn increases the circulation [30]. Considering that the movement in thorax is 3-dimensional, the measurement is difficult to perform, and today the reliability of the technique is unknown. Patients in the present study expressed that the breathing exercises in the yoga intervention had a direct effect on their breathing technique, which is in line with findings in the literature review on the effects of Hatha yoga on musculoskeletal and cardiopulmonary function [30]. The effect of yoga in patients with chronic obstructive pulmonary disease (COPD) has also been demonstrated more recently [15]. 

In this study, no significant difference was found between the yoga group and the control group, regarding the scoring of perceived pain. In both groups, the scoring of pain was decreased at the end of the study. Pain is a complex condition including physical, psychological, and social factors, and it is possible that the intervention in this study was too short to have an effect on pain [13]. In previous studies showing effects on pain, yoga exercises were adapted to a specific pain location [31] or to specific diagnoses [14]. It is also possible that the patients in the control group, as they were aware of being studied, became more observant on their symptoms and well-being and thereby viewed their pain in a new light. The overall extent of individual training was not registered in this study. The patients were not specifically asked to practice the exercises at home between the training occasions and this may be another reason for the lack of significant differences regarding pain between the groups in the present study. In a recent study by Michalsen et al. [32], two yoga programs with 12 respective 24 sessions over 3 months were equally effective in reducing stress, anxiety, depression, and bodily complaints in a group of community-dwelling female volunteers with high levels of perceived stress. The dropout rate at completion of the study was 5%, and this is considered low. This could indicate that this patient group with stress-related symptoms was motivated and suitable for this kind of treatment.

In this clinical context, we found it important that the study design was controlled because yoga is a new treatment in this patient group and setting. However, generalization of the result could be difficult because of the patients' wide range of overlapping symptoms. There was an uneven distribution between men and women in this study, which could be regarded as a limitation for the external validity of the findings. Unfortunately, no stratified randomization for gender was performed. The skewed distribution is nevertheless similar to the distribution of patients seeking medical care for stress-related symptoms in primary health care. On the other hand, Strömberg et al. have shown that the psychosocial stressors, such as feeling very stressed, perceiving poor health, and being dissatisfied with the family situation, are equally associated with depression in men and women [33]. 

The physiotherapist who carried out the assessments was not blind to the participant's group allocation, but since the patients filled in the questionnaire themselves, this was considered acceptable. Furthermore, she was not involved in the yoga intervention. The use of self-rated questionnaires and the fact that the patients were nonblinded to the intervention could be regarded as limitations in this study design. In order to reduce the so-called “Hawthorne effect” (improvement or modification of an aspect simply in response to the fact that the patients know they are being studied), we calculated and compared the mean change from baseline to the end of the study between the groups. However, it is still possible that there is a placebo effect in the yoga group, depending on positive expectations from the patients. An intervention such as the one described is not possible to exactly find out what part of the intervention that had an effect: the yoga, the scheduled activity, or the belonging to a group with others suffering from the same condition. Methodologically, it would have been most appealing to evaluate medical yoga compared to an entirely untreated control group. Due to ethical reasons, the participants in both groups maintained their usual care during the study period. Although, this can be regarded as a study limitation, it would not be ethically acceptable to withdrawthe usual care in order todecrease possible confounders for the study results. 

The outcome measures in the present study were primarily Swedish versions of well-known questionnaires for the measurement of stress-related symptoms such as general stress, burnout, and sleeping disturbances. The PSS is an empirically established index of general stress appraisal, designed to measure the degree to which situations in one's life are appraised as stressful during the last month. All outcome measures were assessed immediately after the 12-week intervention period, but since the PSS refers to stress levels during the past month, we chose to perform this evaluation by telephone interview evaluation by telephone interview one month after the end of intervention. According to Cohen et al., the PSS has adequate reliability and validity also when administered by telephone interview [17]. Furthermore, it has adequate internal and test-retest reliability [17] and has proven internal consistency [18]. 

For the measurement of insomnia, ISI was used. According to Bastien et al. [23], ISI has an adequate internal consistency (Cronbach's *α* 0.74) and is a reliable self-report measure to evaluate perceived sleep difficulties. ISI is as well a valid and sensitive measure to detect changes in perceived sleep difficulties with treatment [23].

Burnout is a mental condition defined as a result of stress exposure. The internal construct validity of SMBQ, which was used for assessment, has been questioned in a recent publication [34]. In a clinical population, seeking medical care at an outpatient stress clinic in Sweden, a revised 18-item version of the SMBQ was shown to satisfy modern measurement standards, when analyzed with confirmatory factor analysis [34]. Chronic burnout has been described to be associated with increased somatic arousal [20]. Prolonged stress may give symptoms of physical, emotional, and cognitive dysfunction such as episodic cognitive problems, tiredness, aches, and pains as well as memory disturbance and difficulties with concentration [2]. Stress-related symptoms and diseases are common in primary health care. As stress-related diagnoses have many over-lapping symptoms, a therapy such as yoga, which takes this complexity into account, may be preferable for this patient group in primary health care. Medical yoga could easily be provided as a group training in the primary health care setting. Long-term evaluations are further needed as well as assessment of the health economic benefits of medical yoga in primary health care. 

## 5. Conclusion

In this randomized controlled study, it was demonstrated that 12 weeks of treatment with medical yoga significantly reduced levels of stress and anxiety and improved the perception of overall health status in patients with stress-related symptoms in primary health care. We conclude that medical yoga is an effective group treatment that is well accepted by patients and can easily be administered in primary health care.

## Figures and Tables

**Table 1:** Demographic data at baseline for the control group and the yoga group.

	Control group (*n* = 19)	Yoga group (*n* = 8)	*P* value
Male/female (*n*)	0/19	3/15	0.06
Age (years ± SD)	52 ± 15	54 ± 9	0.72
Professional (*n*)	8 (42%)	13 (72%)	0.63
Sick leave (*n*)	7 (37%)	3 (17%)	0.17
Retired for reasons of age (*n*)	4 (21%)	2 (11%)	0.41
Years since onset of stress-related symptoms (years ± SD)	9 ± 10	9 ± 6	0.96
Visits to the doctor in the last 8 months (years ± SD)	5 ± 6	7 ± 6	0.44
Visits to the physiotherapist in the last 8 months (years ± SD)	5 ± 6	8 ± 7	0.25
Visits to the counselor in the last 8 months (years ± SD)	3 ± 4	2 ± 4	0.44
Visits to the nurse in the last 8 months (years ± SD)	5 ± 5	5 ± 4	0.66

Data are presented as mean ± SD or number of patients or visits. *P* values refer to the difference between the control group and the yoga group before study start (chi-2-test, unpaired *t*-test, Mann-Whitney *U* test, and *P* < 0.05). SD: standard deviation.

**Table 2:** Stress, burnout symptoms, anxiety, insomnia, and perception of health status at baseline and after 12-week medical yoga intervention.

	Before intervention			After intervention			Difference between study groups regarding change in symptoms	
	Control group	Yoga group	*P* value	Control group	Yoga group	*P* value	Mean difference [95% CI]	*P* value
PSS	34.2 ± 10.7	36.3 ± 10.2	0.749	32.1 ± 8.9 (−2.2)	18.7 ± 6.7 (−17.7)	0.000	15.5 [8.1–23.0]	0.000*
SMBQ	4.7 ± 0.8	4.4 ± 1.2	0.638	3.7 ± 0.6 (−1.0)	3.2 ± 0.6 (−1.1)	0.024	0.1 [−0.4–0.7]	0.412
HADS, total	21.2 ± 8.3	19.9 ± 9.8	0.738	17.2 ± 8.2 (−4.1)	10.6 ± 6.7 (−9.4)	0.014	5.3 [0.0–9.8]	0.047*
HADS, anxiety	12.7 ± 5.0	11.8 ± 5.0	0.680	10.5 ± 4.5 (−2.2)	6.4 ± 3.5 (−5.3)	0.008	3.2 [0.8–5.6]	0.019*
HADS, depr.	8.5 ± 3.9	8.2 ± 5.1	0.903	6.6 ± 4.3 (−1.9)	4.1 ± 3.8 (−4.1)	0.045	2.1 [−0.2–4.6]	0.123
ISI	15.7 ± 4.5	11.4 ± 7.6	0.075	14.1 ± 5.1 (−1.6)	8.0 ± 6.3 (−3.4)	0.004	1.8 [−1.2–4.7]	0.334
EQ-VAS	43.6 ± 20.6	50.7 ± 21.3	0.253	49.2 ± 17.5 (5.5)	69.8 ± 22.9 (19.1)	0.001	−13.6 [−26.0– −1.2)]	0.018*

Data are presented as mean ± SD. *P* values refer to the difference between the control group and the yoga group before, after and for the *difference between study groups regarding mean change in symptoms after the study period. The change in scores was calculated (after intervention and before intervention), as shown within parenthesis. (Mann-Whitney *U* test, *P* < 0.05). 95% confidence interval of the difference between groups is presented. Control group, *n* = 19 and yoga group, *n* = 18. CI: confidence interval; Euro Quality of Life-visual analogue scale (EQ-VAS); HADS: Hospital Anxiety and Depression Scale; ISI: Insomnia Severity Index; PSS: Perceived Stress Scale; SMBQ: Shirom-Melamed Burnout Questionnaire.

**Table 3:** Pain drawing at baseline and after the 12-week medical yoga intervention.

Pain drawing	Before intervention			After intervention		
	Control group	Yoga group	*P* value	Control group	Yoga group	*P* value
Head (*n*)	5 (26%)	5 (28%)	0.920	2 (10%)	1 (6%)	0.580
Neck/shoulders (*n*)	14 (74%)	16 (89%)	0.238	12 (63%)	10 (56%)	0.638
Upper extremity (*n*)	10 (53%)	12 (67%)	0.385	11 (58%)	9 (50%)	0.630
Back/trunk (*n*)	16 (84%)	13 (72%)	0.376	14 (74%)	8 (44%)	0.070
Lower extremity (*n*)	15 (79%)	11 (61%)	0.235	13 (68%)	7 (39%)	0.072

Data are presented as number (%) of patients who reported pain in the actual pain drawing area.

*P* values refer to the difference between the control group and the yoga group (chi-2 test, *P* < 0.05).

Control group, *n* = 19 and yoga group, *n* = 18.

**Table 4:** Oxygenation, heart rate, and blood pressure at baseline and after the 12-week medical yoga intervention.

	Before intervention			After intervention			Difference between study groups regarding change in symptoms	
	Control group	Yoga group	*P* value	Control group	Yoga group	*P* value	Mean difference [95% CI]	*P* value
SaO_2_	96 ± 2	96 ± 2	0.419	96 ± 1	96 ± 1	0.657	−0.7 [−1.9–0.6]	0.289
Heart rate	79 ± 13	74 ± 10	0.206	78 ± 12	71 ± 9	0.067	1.8 [−5.6–9.2]	0.623
Blood pressure, systolic	145 ± 24	139 ± 20	0.402	126 ± 19	132 ± 29	0.513	−11 [−23–0.1]	0.050
Blood pressure, diastolic	89 ± 8	88 ± 12	0.613	82 ± 10	82 ± 12	0.905	−1 [−7.7–5.2]	0.569

Data are presented as mean ± SD. *P* values refer to the difference between the control group and the yoga group (unpaired Student's *t*-test, *P* < 0.05). Control group, *n* = 19 and yoga group, *n* = 18. CI: confidence interval; SaO_2_: peripheral oxygen saturation.
